# Correlates of Inconsistent Refusal of Unprotected Sex among Armenian Female Sex Workers

**DOI:** 10.1155/2014/314145

**Published:** 2014-10-01

**Authors:** Karine Markosyan, Delia L. Lang, Ralph J. DiClemente

**Affiliations:** ^1^Center for Health Services Research and Development, American University of Armenia, 40 Baghramyan Avenue, 0019 Yerevan, Armenia; ^2^Emory University, Rollins School of Public Health, Atlanta, GA 30322, USA; ^3^Emory Center for AIDS Research, Atlanta, GA 30322, USA; ^4^Department of Pediatrics, Division of Infectious Diseases, Epidemiology, and Immunology, Emory University School of Medicine, Atlanta, GA 30322, USA; ^5^Department of Medicine (Infectious Diseases), Emory University School of Medicine, Atlanta, GA 30322, USA

## Abstract

This cross-sectional study assessed the prevalence and correlates of inconsistent refusal of unprotected sex among female sex workers (FSWs) in Armenia. One hundred and eighteen street-based FSWs between the ages of 20 and 52 completed a questionnaire assessing FSWs' demographic, psychosocial, and behavioral characteristics. A total of 52.5% (*n* = 62) of FSWs reported inconsistent refusal of unprotected sex with clients in the past 3 months. Logistic regression analysis controlling for participants' age and education revealed that perceiving more barriers toward condom use (AOR = 1.1; *P* < 0.01), reporting more types of abuse (AOR = 2.1; *P* < 0.01), and setting lower fees for service (AOR = 0.9; *P* = 0.02) significantly predicted inconsistent refusal of unprotected sex. HIV-risk-reduction behavioral interventions tailored to FSWs working in Yerevan Armenia should address the factors identified in this study toward the goal of enhancing refusal of unprotected sex and ultimately preventing acquisition of sexually transmitted infections (STIs) including HIV.

## 1. Introduction

Despite considerable advancement in prevention and treatment, HIV/AIDS remains a major public health concern globally. While in several parts of the world the efforts to contain the epidemic have led to a stabilization of HIV prevalence levels, in transitional societies of post-Soviet Eurasia, the epidemic continues to expand [1]. In Armenia, a country of the former Soviet Union, with a population around 3 million, the number of newly registered HIV cases has been increasing steadily since reporting began in 1988 [2]. However, thus far the country has been spared from a generalized HIV/AIDS epidemic: the estimated adult prevalence in Armenia is 0.2%, and the virus is concentrated in high-risk groups including female sex workers [1–4].

According to UNAIDS report for Eastern Europe, female sex workers (FSWs) are one of the groups at the highest risk for HIV acquisition [1]. Research suggests that, among FSWs in Ukraine, HIV prevalence may be as high as 31.0% [5]. In Armenia, according to recent surveillance, the estimated HIV/AIDS rates among FSWs may be around 1%, representing a prevalence rate that is 5 times higher compared to the general adult population [1, 6]. This disparity represents a disproportionate burden of HIV infection in this population.

Limited information is available on sex work in Armenia. According to studies conducted by UNAIDS in conjunction with Armenia's Ministry of Health, the estimated number of FSWs in the country in 2008 and 2010 varied between 4,000 and 6200 (country population 3.2 million), although the numbers could be as high as 8,100 [7, 8]. Of these, approximately 4,601 (and as high as 6,545) lived in the capital city of Yerevan (city population 1.2 million) [8]. Sex work in Armenia is considered illegal under civil code, but it is not prosecuted by the criminal code. However, pimping, running brothels, or knowingly infecting others with HIV is considered criminal offenses and may lead to prosecution [9]. There are several subgroups of FSWs in Armenia. The largest and most disadvantaged are the “street” sex workers who work individually, without protection from pimps or their peers. They gather in specific locations in the evenings, meet their clients, negotiate specific terms, and go to locations such as hotels, saunas, bathhouses, and bars, to provide services. Street FSWs are the main target of actions implemented by the regulatory and law enforcement agencies. The results of the few studies conducted among Armenian FSWs indicate that they have no other sources of income except for commercial sex work. Furthermore, the majority have minor children and elderly parents for whom they are the only source of income [9–12]. In addition to the aforementioned, Armenian FSWs face increased risk of HIV infection by virtue of their work, through the compounding effects of traditional social norms, stigma, and low HIV knowledge in the general population [13], as well as due to increased seasonal labor migration of FSWs' potential clients to Ukraine and the Russian Federation, where HIV/AIDS rates are high [14, 15].

Given the disproportionate burden of HIV infection among Armenian FSWs and their elevated HIV-risk, a study was conducted in 2003 aimed at identifying behavioral and psychosocial HIV-risk factors and addressing them in future interventions [16]. Based on the results of the study, a 2-hour, one-on-one delivered HIV prevention intervention targeting Armenian FSWs was designed and implemented between 2007 and 2008 [17]. The prevention intervention was predicated on three theoretical models: social cognitive theory, the information-motivation-behavioral skills model, and the theory of gender and power. Thus, perceived social constructs around gender roles, gaps in knowledge surrounding HIV transmission dynamics, motivation to engage in health behaviors, and behavioral skill for condom use and self-efficacy were conceptualized as key modifiable constructs that could increase protective behaviors and guided the overall development of the intervention. Motivational enhancement interviewing, an effective strategy to increase motivation for behavior change, was used to deliver the intervention. A total of 120 street-based FSWs were involved in the study and randomized to either the intervention condition or a wait list control condition. A rigorous randomized controlled trial design was utilized to assess whether the intervention was able to favorably change the HIV-related knowledge, attitudes, and beliefs and ultimately reduce high-risk sexual behaviors among Armenian FSWs. Participants in both conditions were assessed at baseline, prior to randomization to conditions, and at 3- and 6-months following intervention [17].

The data collected at baseline were used to further investigate the HIV-risk and protective behaviors of FSWs which might aid in tailoring future interventions to the needs of the target population. As in HIV-risk-reduction behavioral studies conducted with FSWs in other countries [18–22], the focus of baseline data analysis was on investigating their condom use behavior [23]. However, while condom use is indeed the most important protective behavior, it is often not directly under FSWs' volitional control. Another effective HIV prevention strategy would be refusing to have unprotected sex with male clients.

To the best of our knowledge, no studies to date have investigated refusal of unprotected sex among FSWs. Studies focusing on refusal of unprotected sex by other female populations are also scant. In a study targeting African American adolescent girls, the frequency of unprotected sex refusal was found to be associated with partner communication attributes about sex [24]. Several HIV/STD prevention intervention studies targeting inner city women, women in health-care settings, and women reporting physical abuse by an intimate partner succeeded in increasing refusal of unwanted sex among participants [25–28]. Further, when refusal skills training was incorporated into sexual-risk-reduction interventions with high-risk minority youth, including African American women, a decrease in HIV-risk behaviors resulted [29–32].

Considering the potential role of refusal skills in decreasing risk behavior, it is important to understand factors that hinder consistent refusal of unprotected sex, particularly among high-risk populations such as FSWs. This study assessed the relation between inconsistent refusal of unprotected sex and demographic, psychosocial, and behavioral factors with the purpose of identifying potential targets for future interventions. We hypothesized that history of abuse, perceived barriers towards condom use, and alcohol consumption during sex work would contribute to inconsistent refusal of unprotected sex as is the case with inconsistent condom use and inconsistent condom application [16, 23, 33]. In addition, we assessed whether refusal behavior is influenced by another sex-work-related factor, fees for service, which was shown to be associated with consistency of condom use among FSWs in Ghana [21].

## 2. Methods

### 2.1. Participants

The study was conducted in Yerevan, the urban capital of Armenia. Between August 2007 and July 2008, FSWs were recruited, using multiple outreach strategies, to participate in a randomized controlled trial of an HIV-risk-reduction intervention designed to reduce the risk of HIV transmission through heterosexual relationships [17]. Recruiters screened 168 self-identified FSWs. Eligibility criteria were being female, 18 years of age or older, trading sex for money in the past 7 days, and cognitively able to participate in the study (based on recruiter's judgment during interaction with potential participant). One hundred and twenty women agreed to participate, which resulted in a 76% participation rate. Women were compensated $20 for their participation. Additional incentives included availability of a physician and an attorney to answer questions about sexually transmitted infections and legal/human rights issues, respectively. Due to economic constraints and sex-work-related stigma, access to such professionals is often prohibitive for this population. Childcare was also made available at the study site. The Institutional Review Boards (IRB) of the American University of Armenia (Yerevan, Armenia) and Emory University (Atlanta, GA, USA) approved the study protocol prior to implementation [17].

### 2.2. Study Design and Data Collection

This study employed a cross-sectional design using the baseline data from the randomized controlled trial of an HIV-risk-reduction behavioral intervention [17]. The data were collected prior to randomization of participants to intervention and control conditions. Baseline assessment consisted of a face-to-face interview administered by a trained interviewer in a private room. Interviews were conducted in Armenian and were approximately 30 minutes in duration.

### 2.3. Outcome Measure

The outcome of the study was self-reported inconsistent refusal of unprotected sex based on an assessment of the frequency with which FSWs refused to engage in penetrative intercourse with a male client without using a condom in the past 3 months. Response categories ranged from 0 (never) to 3 (every time) and responses were dichotomized such that FSWs who reported refusing unprotected sexual intercourse with a male client “every time” were considered “consistent refusers” while all others were considered “inconsistent refusers.”

### 2.4. Predictor Measures

Sociodemographic and sex-work-related variables were assessed including age, education, marital status, having children, frequency of alcohol consumption during sex in the past 7 days, and the amount of money charged for condom-protected vaginal sex. The constructs for potential psychosocial predictors of unprotected sex refusal (e.g., barriers toward condom use and abuse experiences) were assessed using theoretically derived and validated measurement scales [17, 29, 30]. Abuse experiences were assessed by three dichotomous items inquiring about participant's lifetime experience of physical, emotional, and/or sexual abuse. A total score was obtained with values ranging between 0 and 3. Barriers to condom use were measured using an 18-item scale assessing attitudes and beliefs that impede participants' ability to effectively use condoms. Sample items included “I wouldn't know where to get a condom” and “Condoms spoil the mood.” Response options ranged from 0 (strongly disagree) to 3 (strongly agree). A total scale score was obtained with values ranging between 0 and 54. The internal consistency of the scale (Cronbach's *α*) was 0.82.

Several techniques were used to enhance the validity of participants' self-reported sexual behavior and other variables. Participants were asked to report their behavior over a relatively brief time interval to enhance accurate recall and were provided calendars specifying the reporting intervals of interest [30]. To enhance confidentiality, interviewers assured participants that codes rather than names would be used on all records.

### 2.5. Statistical Methods

Descriptive statistics were computed to summarize sociodemographic characteristics of the entire sample. Next, to identify potential covariates for inclusion in the regression model, differences between consistent and inconsistent refusers were analyzed with regard to sociodemographic and other psychosocial and behavioral variables. Chi-square and independent samples *t*-tests for dichotomous and continuous variables, respectively, were performed. Finally, a logistic regression model was constructed to identify significant predictors associated with inconsistent refusal of unprotected sex, controlling for covariates in the model. Variables were selected for inclusion in the model if they were statistically significant at *P* ≤ 0.20 in bivariate analyses [34] or were theoretically or empirically identified as potential confounders (e.g., age and education). Analyses were performed using PASW 21 statistical software.

## 3. Results

### 3.1. Descriptive and Bivariate Analyses

A total of 120 FSWs between the ages of 20 and 52 (mean = 33.7; SD = 6.7) comprise the sample. Of these, 118 provided usable data for this analysis, representing those who reported experiencing a male client who wanted to have sex without a condom when the participant wanted to use a condom. Two FSWs who reported that this has never happened to them were removed from analyses, leaving an analytic sample of 118 women. Of the 118 participants, 58.5% (*n* = 69) completed 10 years of education or more and a total of 17.8% (*n* = 21) reported being single (never married), while others reported being either divorced or widowed, married, or unmarried but living with a man. Among all participants, 62.7% (*n* = 74) reported having children, and 47.5% (*n* = 56) reported living with their children. A total of 57.6% (*n* = 68) participants reported a history of physical, emotional, and/or sexual abuse with a mean abuse score of the sample 1.1. Further, the strongest barriers to condom use in this study included anticipation of negative attitudes of clients toward using condoms. For example, 26% of participants thought that if they asked their clients to use a condom, clients might get turned off. Nearly one-third of participants (*n* = 35) thought that if they asked their clients to use a condom, clients might get angry. The proportion of FSWs reporting inconsistent refusal of unprotected sex in the past 3 months was 52.5% (*n* = 62) including the respondents who reported having refused unprotected sex “sometimes” (32.2% (*n* = 38)), “rarely” (18.6% (*n* = 22)), or “never” (1.7% (*n* = 2)). A total of 47.5% (*n* = 56) FSWs reported consistently (every time) refusing unprotected sex.

Additional descriptive data as well as bivariate analyses are presented in Table 1. Differences between inconsistent and consistent refusers that reached significance at *P* ≤ 0.20 in bivariate analysis were observed with respect to experiences of abuse, barriers toward condom use, and fees for service. There was no statistically significant difference between groups with respect to the frequency of alcohol use during sex work in the 7 days preceding the assessment; therefore, this variable was not used in subsequent analyses.

### 3.2. Regression Analysis

Table 2 presents the results of the logistic regression analysis with inconsistent refusal of unprotected sex as the outcome variable. Of the 5 variables entered into the model, three had statistically significant association (*P* ≤ 0.05) with the outcome. The significant predictors of inconsistent refusal of unprotected sex included a higher number of barriers toward condom use (AOR = 1.1; *P* < 0.01), a higher number of different types of abuse experienced by FSW (AOR = 2.1; *P* < 0.01), and lower fees for service (AOR = 0.9; *P* = 0.02). Specifically, with each unit increase in perceived condom use barriers, FSWs were 10% more likely to report inconsistent refusal of unprotected sex. Additionally, with a unit increase in the number of abuse experiences, FSWs were 210% more likely to report inconsistent refusal of unprotected sex. Finally, with every 1000 AMD ($2.60) increase in service fee per client, FSWs were 10% less likely to report inconsistent refusal of unprotected sex.

## 4. Discussion

To the best of our knowledge, this is among the first studies to assess prevalence of and factors associated with inconsistent refusal of unprotected sex among FSWs. The findings suggest that among Armenian FSWs the prevalence of that risk behavior is high: a total of 52.5% (*n* = 62) study respondents reported having practiced it in the past 3 months. Given the high risk for HIV/STI transmission among FSWs and the importance of consistent refusal of unprotected sex as a preventive strategy, the high prevalence of inconsistent refusal is concerning and must be addressed by rigorous public health research.

Based on the results of the regression analyses, our hypothesis regarding the association between refusal of unprotected sex on one hand and experiences of abuse and barriers toward condom use on the other hand was supported. The results of the regression analysis also showed an association between refusal of unprotected sex and the fees charged per client or service. Findings of the current study suggest that FSWs, who inconsistently refuse unprotected sex, can be characterized as those who have experienced more types of abuse, have more impediments to condom use, and charge lower fees for service.

Abuse is prevalent among disempowered female populations globally [35–41], and Armenian FSWs are no exception. It is not surprising, therefore, that fear of past and possibly current abuse may lead to disempowerment such that FSWs more frequently engage in HIV-risk sexual behaviors and are less likely to refuse unprotected sex. The association between abuse and inconsistent refusal of unprotected sex found in this study corroborates findings of prior studies having shown that abuse was associated with various HIV-risk factors including unwanted sex, inconsistent condom use, condom failure, and STI symptoms [23, 35–43]. Abuse is a multifaceted public health issue that is complex and challenging to address. Nevertheless, a multilayered violence intervention in India targeting policy makers, secondary stakeholders (police, lawyers, and media), and primary stakeholders (FSWs), as part of a wider HIV prevention programming, succeeded in reducing experiences of violence among participants [44]. Also, a gender-specific HIV/STD prevention intervention among women reporting physical abuse by a current or recent (past year) intimate partner resulted in decreased unprotected sex occasions, increased usage of an alternative strategy (e.g., refusal, “outercourse,” or mutual testing) and having a safer sex discussion with their partners [28]. Therefore, services targeting FSWs in Armenia should incorporate a screen for history of violence in an effort to provide needed support and resources to affected women offering safe refuge from abusive relationships and assistance in recovery from traumatic experiences.

The correlation of a higher number of perceived barriers toward condom use with inconsistent refusal of unprotected sex is also supported by previous studies showing similar associations between perceived barriers and other HIV-risk behaviors, such as inconsistent condom use and inconsistent condom application [23, 33]. This association might be explained by the finding that the strongest barriers to condom use in this study included anticipation of negative attitudes of clients toward using condoms, for example, fear that if FSWs asked their clients to use a condom, clients might get turned off or get angry. Having such beliefs in addition to fear of abuse, a FSW is less likely to negotiate condom use or to actually refuse sex without a condom. These perceptions are modifiable and they should be addressed in behavioral interventions.

The association between lower fees for service and inconsistent refusal of unprotected sex is also corroborated by a study demonstrating a correlation between lower fees for service and inconsistent condom use among FSWs in Ghana [21] as well as by studies indicating that the majority of Armenian FSWs have no other source of income except for commercial sex work and have children and parents for whom they are the only support [9–12]. FSWs who have not established a repeat client list, whose services are less frequently requested by clients, and who are not able to set higher fees may feel that they have less freedom to deny services. Further data is needed to elucidate this association; however, this may suggest that disadvantaged FSWs should be the primary focus of tailored interventions.

The study has several implications. First, interventions targeting FSWs must address the reality of abuse and experiences of abuse both didactically and through proper support and assistance. Learning to identify abusive situations and reducing stigma associated with being a victim of abuse as well as knowing where to seek help are crucial aspects of addressing this complex issue. Further, interventions should specifically address and enhance FSWs' skills to motivate clients to desire to use condoms as a pleasurable aspect of sexual intercourse as well as out of concern for their own safety. Finally, effective communication skills to negotiate condom use as well as refuse sex without condoms must be a core component of any intervention targeting this population. Such skill building approaches have been found to be significant in helping other at-risk populations to reduce their vulnerability to HIV/STI acquisition [25, 29–32, 45].

The current study has several limitations. This is a cross-sectional study with a relatively small sample size. Recruitment was limited by the outreach efforts of a local nongovernmental organization, Hope and Help, and convenience sampling techniques, possibly introducing a selection bias that would limit generalizability. Compared to the national FSW surveillance data, participants in the current study were older (33.7 versus 30.1), reported older age of first commercial sex (25.6 versus 22.0), and had greater prevalence of divorce (66.7% versus 38.8%) [12]. These variables have been associated with sexual-risk behavior in other FSW subpopulations [18–21]. Future efforts could use additional waves of respondent driven sampling to reach diverse FSW subpopulations and increase generalizability.

The current study also lacked validation of self-reported behaviors through STI testing. Despite our efforts to minimize social desirability bias, due to the nature of the future intervention, participants were aware that refusing unprotected sex is a healthy behavior and may have wished to report higher rates. Despite efforts to minimize social desirability bias, data suggest that participants in this study likely under reported socially undesirable behaviors including drug use (0%) as well as oral (8.3%) and anal sex (0%). These rates were considerably lower than corresponding rates obtained from the national surveillance survey (9.3%, 27.3% and 40.1% resp.) [12], suggesting the presence of social desirability bias.

## 5. Conclusions

Despite mentioned limitations, as one of the first studies to investigate an important HIV-risk behavior, inconsistent refusal of unprotected sex with male clients, the present work could open the way to future studies on this issue among FSWs in Armenia as well as in other countries with similar environments. While condom use is widely accepted as the best form of protection, existing dynamics between FSWs and their clients may often preclude use of condoms. Therefore, it is imperative that interventions targeting FSWs address refusal of unprotected sex as an effective protective strategy and address the skills necessary to empower women to implement this protective behavior in an effort to avoid the transmission of STIs, including HIV, among FSWs and their clients in Armenia.

## Figures and Tables

**Table 1:** Descriptive statistics and bivariate associations between inconsistent and consistent refusers of unprotected sex in the past 3 months.

Variable	Inconsistent refusers (*n* = 62)	Consistent refusers (*n* = 56)	*P*
	Mean (SD) % (*N*)	Mean (SD) % (*N*)	
∗Age	33.5 (7.3)	34.0 (6.1)	0.64
∗Education (≥10 years)	59.7% (37)	57.1% (32)	0.78
Marital status (single)	21.0% (13)	14.3% (8)	0.34
Having a steady partner	50.0% (31)	50.0% (28)	1.0
Having children	62.9% (39)	62.5% (35)	0.96
Number of clients in 7 days	6.1 (5.3)	5.8 (5.1)	0.72
∗Fees for service^1^	9.4 (5.4)	11.9 (6.2)	0.02
∗Types of abuse	1.5 (1.2)	0.6 (0.8)	<0.01
Alcohol consumption	2.5 (3.2)	1.9 (4.1)	0.39
∗Barriers to condom use	22.2 (8.1)	15.4 (7.8)	<0.01

*Variables included in the logistic regression model.

^1^Fees for service in thousands AMD (Armenian currency) (1,000AMD = $2.6).

**Table 2:** Logistic regression model.

Predictors/covariates	AOR^1^ [95% CI^2^]	*P*
Age	1.0 [0.9–1.1]	0.58
Education (≥10 years)	1.3 [0.5–3.3]	0.57
Fees for service	0.9 [0.8–1.0]	0.02
Experiences of abuse	2.1 [1.4–3.3]	<0.01
Barriers to condom use	1.1 [1.0–1.2]	<0.01
Constant	0.5	0.67

^1^AOR: adjusted odds ratio using consistent refusal of unprotected sex as the reference category.

^2^CI: confidence interval.
